# UV-Induced Skin Cancer Knowledge, Sun Exposure, and Tanning Behavior among University Students: Investigation of an Opportunity Sample of German University Students

**DOI:** 10.1155/2021/5558694

**Published:** 2021-12-29

**Authors:** Marc Rocholl, Julia Hannappel, Michaela Ludewig, Swen Malte John

**Affiliations:** ^1^Institute for Health Research and Education, Department of Dermatology, Environmental Medicine and Health Theory, University of Osnabrück, Am Finkenhügel 7a, Osnabrück 49076, Germany; ^2^Institute for Interdisciplinary Dermatological Prevention and Rehabilitation (iDerm), University of Osnabrück, Am Finkenhügel 7a, Osnabrück 49076, Germany

## Abstract

Ultraviolet radiation (UVR) is the most important risk factor for developing skin cancer. University students can be considered as a particularly high-risk group for long- and short-term adverse effects of UVR due to intensive solar UVR exposure and high rates of sunburn. While validated questionnaires for assessing solar UVR exposure and sun protection behavior are available in German, a questionnaire for assessing the level of knowledge about this topic is still missing. We conducted a literature search for cross-sectional studies assessing skin cancer and sun protection knowledge among university students in Medline (via PubMed) and analyzed existing questionnaires and topics contained therein. We chose to translate the “Skin Cancer and Sun Knowledge Scale” referring to the TRAPD method into the German language and pilot-tested the translation with an opportunity sample of German students. The literature search revealed 36 eligible studies. Four major topics were identified within the studies: knowledge on skin cancer, risk factors, UVR, and sun protection measures. One hundred and seven German university students (86.0% female) with a mean age of 26.25 years (SD ± 4.58; range: 19–46) participated in our pilot study. The internal reliability of the scale was KR-20 = 0.624. We discovered an improvable level of knowledge in terms of skin cancer among the study population. Statistical analyses revealed no significant associations between the level of knowledge and UVR exposure or tanning behavior, respectively. The skin cancer and sun protection knowledge of German university students should be examined thoroughly. While the psychometric properties of the SCSK require further thorough investigation, first empirical experiences indicate the suitability of the tool to assess the level of knowledge regarding skin cancer and sun protection.

## 1. Introduction

Skin cancer is one of the most common types of cancer worldwide within the Caucasian population and therefore a significant public health issue [1–4]. In the past decades, malignant melanoma (MM) and keratinocyte carcinoma (KC, or nonmelanoma skin cancer (NMSC)) are increasing rapidly in incidence across the globe [1–5]. More than 300,000 cases of MM were detected in 2017, leading to approximately 62,000 global deaths [6]. In addition, there were 7.7 million new cases of KC annually throughout the world, 5.9 million due to basal cell carcinoma (BCC) and 1.8 million due to squamous cell carcinoma (SCC) [6]. The most significant risk factor for developing skin cancer is ultraviolet radiation (UVR) [7]. In 1992, the *International Agency for Research on Cancer* (*IARC*) has already classified solar radiation as carcinogenic (Group 1) and UVR as probably carcinogenic to humans (Group 2A) [8]. In 2009, UVR was reassessed and has also been classified as carcinogenic to humans (Group 1) [9]. Besides long-term effects of solar radiation, short-term effects—such as sunburns (erythema)—are also of relevance as, for example, increased sun exposure during childhood, especially if leading to sunburns, increases the risk of developing melanoma [7, 10–12].

Almost all adverse effects of solar UVR exposure are preventable by adopting adequate sun-protective measures. Prevention strategies mainly aim at reducing solar UVR exposure, in particular by limiting outdoor exposure during peak solar UVR hours (regionally depending on solar noon, approximately between 11 a.m.–4 p.m.), avoiding tanning beds, seeking shade whenever possible, and applying sun protection measures, which include wearing long‐sleeved shirts and trousers, wearing a wide-brimmed hat, wearing sunglasses, and applying sunscreen [13, 14]. Due to the long latency period of some adverse effects, it is of particular importance to implement sun-protective measures early as well as permanently. However, especially adolescents and young adults are often exposed to high levels of solar or artificial UVR (e.g., sunbeds), and studies show high rates of sunburn and inadequate protection behavior [15, 16]. In addition, outdoor tanning is still popular among the aforementioned age groups as a tan is perceived as attractive and as a sign of good health in many Western countries [13, 17]. As various studies (among others [18–23]) point out, university students can be considered as a particularly high-risk group for long- and short-term adverse effects of UVR—due to a high solar UVR exposure, insufficient application of sun-protective measures, and positive attitudes toward tanning—and therefore should receive particular attention.

While the importance of knowledge in predicting behavior has been widely discussed in various health contexts [24], the body of evidence regarding sun-protective behavior is still inconclusive. In this regard, Nahar et al. [25] highlight deficiencies in terms of sunscreen and UVR knowledge, a moderate-to-high level of skin cancer knowledge, but also an insufficient use of sun-protective measures among medical students from Australia, Brazil, Peru, Albania, Canada, England, France, Hungary, Malaysia, Pakistan, Poland, Romania, Sweden, Turkey, and the USA. These findings are in accordance with studies reporting an overall satisfactory level of knowledge in various student samples (e. g., nursing, osteopathic, and medical students) in the USA, Turkey, Saudi Arabia, Spain, and Ireland [18–20, 26–29]. Nevertheless, this solid level of knowledge does not necessarily seem to be associated with adequate sun-protective behavior [18, 19, 27–29]. This is particularly of interest, because health behavior approaches assume that knowledge about health hazards seems to act a part in the development of attitudes—especially risk perceptions—and thus may have an (implicit) influence on the initiation of behavioral change [30].

Although solar UVR exposure and sun-protective behavior as well as the level of knowledge about skin cancer and sun protection are intensively investigated among various student populations in other countries, little is known about German university students. To provide reliable data on exposure as well as behavior and knowledge, validated questionnaires are needed. While the “*Sun Exposure and Protection Index*” (SEPI) [31], which is a validated questionnaire for the assessment of solar UVR exposure and sun protection behavior has recently become available in German, a questionnaire for assessing the level of knowledge is still missing. As Day et al. [32–34] emphasize, differences in the measurement of knowledge should be avoided to facilitate the comparison of findings between studies. Before an investigation of German students is conducted, a questionnaire to capture the level of knowledge in terms of skin cancer and sun protection adequately should be developed.

The aims of the present study therefore were (1) to provide an overview of the questionnaires used to assess knowledge regarding skin cancer and sun protection among university students and the topics contained therein and, based on this, (2) to select a suitable questionnaire, to translate and pilot-test it with an opportunity sample of German students. Furthermore, we aimed for (3) assessing students' sun exposure and outdoor tanning behavior.

## 2. Materials and Methods

### 2.1. Part I—Literature Search

To provide an overview on questionnaires assessing knowledge in terms of skin cancer and sun protection, we performed a literature search in Medline (via PubMed) using the search terms “*knowledge AND (skin cancer OR sun protection) AND student^∗^*.” We conducted the literature search until December 31, 2018, and updated our search lastly on January 21, 2021. Search was performed without any limitations or filters. We included cross-sectional studies reporting the assessment of knowledge among any kind of university students. Non-English language papers were excluded. After examining titles and abstracts for eligibility, the full texts of potentially eligible studies were subsequently reviewed and checked for inclusion suitability. We extracted study characteristics (e.g., country, setting, study sample) and topics of the used questionnaires. If further literature was referenced regarding the questions and topics included, we reviewed these and extracted the topics as needed. Afterward, a content analysis with theory-driven and inductive approaches was used to systematize major themes across questionnaires.

### 2.2. Part II—Translation and Pilot Testing of the Questionnaire

The translation of the chosen questionnaire was performed referring to the TRAPD method, which has been issued in numerous publications and guidelines (e. g., the European Social Survey (ESS) Translation Guidelines or the GESIS Survey Guidelines) [35–38]. The TRAPD method requires two independent translations of the same questionnaire (*T* *=* *translation*). During the translation process, both translators make sure that the translation is correct and complete in terms of content and language, and that the language is easy to understand and suitable for the target group. In addition, the key terms of the respective topic should be used consistently. As a next step, a reviewer compares the translations together with the translators and discusses and clarifies questions regarding their translations (*R* = *review*) to develop a joint final version (*A* = *adjunction*). In the following pretest, the translated questionnaire is tested on a small sample of the target group (*P* = *pretest*). Documentation occurs during the entire process (*D* = *documentation*) to ensure that the development of the final version remains comprehensible [35–38]. In our case, the translators discussed their translation with each other, so that we modified the TRAPD method regarding the steps review and adjunction at this point. Subsequent to the systematic translation process described above, we conducted a pretest with four students to eliminate any comprehension difficulties.

#### 2.2.1. Design of the Pilot Test

We pilot-tested our translation within a small cross-sectional study at the University of Osnabrueck, Germany, in May 2019 with an opportunity sample of students of high-level vocational education teaching, enrolled in the summer term of 2019. Data collection was carried out by means of a written questionnaire distributed in two lectures and five seminars. Students not studying vocational education teaching or occasional students were excluded. At the beginning of each survey, the study objectives were presented and information about voluntary participation and anonymous collection, use, and analysis of data was given. All participants agreed to participate voluntarily. The students were informed that withdrawal of approval at any time would not lead to any negative consequences. In this way, informed consent was ensured. The survey and analysis of the data were conducted in accordance with the principles of the *General Data Protection Regulation* (*GDPR*) [39].

#### 2.2.2. Measures

The questionnaire was divided into three parts. At first, we assessed sociodemographic data, such as age, sex, personal or family history of skin cancer, and the self-assessed skin phototype according to the Fitzpatrick skin phototype classification system [40]. Furthermore, the participants were asked about their previous vocational training, study course, and current study program.

Moreover, the students' *sun exposure* was assessed according to Glanz et al. [41] by means of two items which capture the average frequency of hours participants spent outdoors per day on weekdays (Monday till Friday) and on weekends (Saturday and Sunday) between 10 a.m. and 4 p.m. in summertime. To that end, the students classified themselves according to a 7-point Likert scale ((1) “*30 minutes or less,*” (2) “*31 minutes to 1 hour,*” (3) “*2 hours,*” (4) “*3 hours,*” (5) “*4 hours,*” (6) “*5 hours,*” and (7) “*6 hours*”). To calculate the average daily sun exposure, the two values were combined and weighted (factor 5 for weekdays and factor 2 for weekends). Thus, between 1 and 7 points could be achieved, so that a higher value indicated a higher average sun exposure per day [34, 41]. Similar to Day et al. [34], the students' outdoor *tanning behavior*—defined as exposing one's body to direct solar UVR in order to tan the skin—was determined by using an item which interrogated the frequency of sunbathing by means of a five-level rating scale ((1) “*Never*,” (2) “*Once or twice a year*,” (3) “*3*–*5 times a year (i.e., once every month or two on average)*,” (4) “*7–12 times a year (i.e., almost every month on average)*,” and (5) “*More than once a month*”). To adequately capture the students' skin cancer and sun protection knowledge, we used the “*Skin Cancer and Sun Knowledge Scale*” (see below) [34].

#### 2.2.3. Statistical Analysis

All data were analyzed using *IBM® SPSS® Statistics 25*. In order to describe the students' characteristics, descriptive statistics were generated. Internal reliability was evaluated by using Kuder-Richardson 20 formula (KR-20). For exploratory analyses between the level of knowledge and sun exposure, the correlation coefficient of Pearson's *r* was computed. We used Spearman's rho to calculate the correlation between knowledge and tanning behavior. Statistical significance was set at *p* ≤ 0.05.

## 3. Results

### 3.1. Part I—Overview of the Literature

The initial literature search revealed 282 results, of which 24 met inclusion criteria. Hand search revealed another four eligible studies. We completely rerun our literature search, lastly on January 21, 2021. Out of the 333 results, 30 (24 from the former search and six new references) met the inclusion criteria. Hand search revealed another two eligible references. The included studies were published between 1993 and 2020 and were conducted in the following countries: Australia (1x), Bosnia and Herzegovina (1x), Brazil (4x), China (1x), France (1x), Iran (1x), Iraq (1x), Ireland (1x), Malaysia (1x), Pakistan (1x), Peru (1x), Poland (2x), Saudi Arabia (2x), Spain (2x), Turkey (4x), and USA (10x). Study characteristics, including details on sample size, study participants, and settings, are reported in Additional File 1.

#### 3.1.1. Overview of Questionnaires

The analysis of the questionnaires used in the included studies revealed four major topics: (1) knowledge on skin cancer, including epidemiological aspects (e.g., prevalence), symptoms or signs of skin cancer (e.g., changing moles) as well as questions regarding lethality or cure rate (e.g., therapy, but also early detection); (2) risk factors associated with skin cancer, especially solar or artificial UVR exposure, sunburn, indoor or outdoor tanning as well as genetic aspects such as heredity or skin type; (3) knowledge about UVR, especially regarding peak solar UVR hours, general knowledge (e.g., about the UV index), but also about further positive and negative effects of solar or artificial UVR (e.g., vitamin D metabolism or photoaging); and (4) knowledge of sun protection measures as well as detailed knowledge on sunscreens and their proper application.  Table 1 provides an overview of the major topics and subtopics included in the questionnaires. It becomes apparent that no questionnaire covers all topics. Most studies did not use validated instruments to measure participants' level of knowledge regarding skin cancer and sun protection knowledge. Most studies fail to report on a systematic approach to question selection or questionnaire development, as knowledge was not always the primary outcome of the study. Based on these results and a discussion within the research group of this work, we found the “*Skin Cancer and Sun Knowledge Scale*” (*SCSK*) to be the most appropriate instrument to capture the level of knowledge and that translation and pilot testing of the instrument might be worthwhile. Therefore, the SCSK was translated into German and used subsequently.

#### 3.1.2. Skin Cancer and Sun Knowledge Scale

The questionnaire was developed by Day et al. [34], based on a systematic review of studies assessing skin cancer and sun protection knowledge among various populations [33]. The SCSK comprises 25 items covering the following five topics: sun protection, tanning, risk factors for skin cancer, prevalence of skin cancer, and symptoms of skin cancer. While the first 15 items are true-false statements, the subsequent 10 items are phrased as multiple-choice questions of which only one answer is correct. Due to the one-factor structure of the SCSK, up to 25 points can be achieved. A higher score indicates better knowledge about skin cancer and sun protection [34]. The psychometric characteristics of the SCSK were tested with a sample of 514 undergraduate students of the University of Adelaide, Australia, and revealed acceptable psychometric properties (internal reliability: KR-20 = 0.69; test-retest reliability: *r* = 0.83, *n* = 52, *p* < 0.001) [33]. Besides the original version of the questionnaire, a Turkish translation was validated by Haney et al. [63] with a sample of nursing students. The results showed less clear but still acceptable psychometric properties (internal consistency: KR-20 = 0.51; 2-week test-retest reliability: *r* = 0.52, *n* = 34, *p* < 0.001) [63].

### 3.2. Part II—Pilot Testing

One hundred and seven students (86.0% female) with an average age of 26.25 years (SD ± 4.58; range: 19–46) were included. The majority of the study participants reported to fall under skin phototype II (50.5%) or skin phototype III (31.8%) according to the Fitzpatrick skin phototype classification system [40]. None but one of the participants reported to fall under skin phototype I or skin phototype V, respectively. The majority of the students completed an apprenticeship prior to their studies. Demographic data on participants enrolled in the study are shown in Table 2.

#### 3.2.1. Descriptive Results

Mean values of sun exposure and frequency of tanning behavior are presented in Table 3. Most students reported to intentionally tan three to five times a year (29.0%, *n* = 31). Twenty-seven students (25.2%) exposed themselves seven to twelve times a year, whereas twenty-five participants (23.4%) stated to tan once or twice a year. A total of twenty-four students (22.4%) stated that on average they consciously exposed themselves to the sun more than once a month to get a tan. No student claimed to never tan.

#### 3.2.2. Level of Skin Cancer and Sun Protection Knowledge

The mean SCSK score of the 107 students was 15.64 (SD ± 3.16; min: 7; max: 23) with a slightly better knowledge among female students (*M* = 15.85; SD ± 3.20 vs. *M* = 14.33; SD ± 2.69). The number of correctly answered items of the SCSK is shown in Table 4. It is particularly striking that merely 10% of the participants knew that a tan is a sign of incipient skin damage.

### 3.3. Statistical Analyses

#### 3.3.1. Internal Reliability

The internal reliability of the scale is KR-20 = 0.624. Even though this is below the value of 0.7, which is generally considered acceptable, it is still appropriate. Analyses show that the internal reliability can be improved slightly to KR-20 = 0.663 by removing items 5, 6, 17, 21, and 22.

#### 3.3.2. Exploratory Statistical Analyses

There was no significant correlation between knowledge and tanning behavior in the investigated sample (*p*=0.247). Furthermore, the level of skin cancer and sun protection knowledge was not significantly associated with sun exposure among the participants (*p*=0.144).

## 4. Discussion

This study aimed to provide an overview of questionnaires used to assess skin cancer and sun protection knowledge among university students. We additionally intended to identify a suitable questionnaire for subsequent translation into German and conducted pilot testing. Furthermore, students' sun exposure and outdoor tanning behavior should be investigated. The SCSK was characterized by a diverse thematic orientation and a combination of multiple-choice questions as well as the true-false statements. In our pilot study, we found reasonable internal reliability of the German translation of the SCSK. The level of knowledge among our sample seems to be improvable. Statistical analyses revealed no significant associations between level of knowledge and solar UVR exposure or tanning behavior, respectively.

Our literature search retrieved a large number of existing studies in which a broad range of different questionnaires were used. The topics therein, however, were heterogeneous; the scope of the surveys on knowledge frequently covered only a very selected portion of the relevant items, related to the specific cohorts the studies were targeting, thus making the results less comparable. The SCSK does also not cover all the topics mentioned in Table 1, despite being based on a systematic review [33], so the content validity needs further discussion. The topics of the scale should therefore be reviewed regarding completeness and relevance. This also applies to the removal of items for reliability reasons. The internal reliability in the present study remained below the generally acceptable value of 0.7. However, it has to be taken into account that knowledge in terms of skin cancer and sun protection, as can be seen from the various topics in the questionnaire, is a heterogeneous construct, which might be an explanation for the low internal reliability. Further research should therefore focus primarily on the content validity of the scale, while reliability (e.g., by conducting test-retest analysis) also has to be taken into account. Subsequently, the scale can be shortened and adjusted, if necessary.

In our study, we assessed the level of knowledge among a small opportunity sample of German students. To capture knowledge among Turkish language populations, Haney et al. [63] validated a Turkish version of the SCSK. Remarkably, they reported an almost entirely equal mean level of knowledge assessed by the SCSK within their sample with a mean value for male students of 14.19 (SD ± 3.05, *n* = 67) and a mean value for female students of 15.28 (SD ± 2.74, *n* = 309). In addition, the two questions regarding gradual tanning (item 8; correctly answered by 29.5%) and the most common skin cancer (item 24; correctly answered by 13.3%) were both incorrectly answered quite often in our study as well as in the study by Haney et al. [63]. The greatly similar findings of Haney et al. [63] might indicate the suitability of the tool in terms of reliability to assess the level of knowledge—also and especially in non-English-speaking countries.

In the present study, we found a slightly higher level of knowledge in female students. Several studies indicate that the overall level of knowledge among women in the general population is higher compared to men. Female predominance in sun protection knowledge has also been observed within other student populations, e.g., in the USA or Turkey [26, 28, 63]. Nevertheless, this still does not necessarily lead to adequate sun-protective behavior [68–72].

Our findings further revealed a clear knowledge deficit among students, especially regarding the assumption that being tanned is not a skin damage (item 9). Several studies, e.g., from Sweden, USA, or Canada [17, 25, 26, 73], showed a high cosmetic value toward tanned skin among students—especially in females—and the misconception that tanned skin is healthy. These findings argue for addressing the aspect of the significance of appearance in educational interventions by using appearance-based approaches, such as UV photography of the skin, revealing skin damage (solar lentigines) yet invisible to the naked eye [74]. Focusing not exclusively on knowledge transfer, but on target group-relevant aspects might result in improved sun-protective behavior in this target group of multipliers [74–76]. At this point, however, it must be noted that tanned skin is considered attractive mainly in Western countries. This is, of course, not the case in all countries of the world as cultural norms may greatly influence views on tanned skin. As a study from South Africa shows, preferences for lighter skin leading to skin-lightening practices are common [77]. Furthermore, migration and cultural adaptation might influence tanning behavior of persons with a migration background (e.g., persons with Asian background), so that across-the-board statements do not always depict reality [78].

In our study, we surveyed the knowledge among students of vocational education teaching and training from different disciplines (i.e., health sciences, nursing sciences, and cosmetic sciences). For future skin cancer prevention efforts, this target group is of particular importance, as they will hold a key role as multipliers in their later professional working life as vocational school teachers [79–81]. These teachers lecture otherwise hard-to-reach target groups who are working in areas potentially related to skin (cancer) care and prevention.

### 4.1. Strengths and Limitations

We performed a literature search exclusively in Medline (via PubMed), which may have led to a lack of sensitivity and to potentially eligible studies being overlooked. Furthermore, our sample size is comparatively small. Moreover, the main focus of our work was on the process of choosing, translating, and pilot testing a validated questionnaire to assess the level of skin cancer and sun protection knowledge. It was not the aim to carry out complex statistical analyses. The statistical analyses in terms of reliability should therefore also be interpreted with caution. Additionally, we pilot-tested the questionnaire by means of an opportunity student sample. Our sample therefore can hardly be considered representative as the percentage of male students in our study was very low. This limits the transferability of our results to German university students in general. Tanning behavior and sun exposure were surveyed retrospectively, so a recall bias may also have influenced our results. Despite the fact that the tanning behavior was surveyed according to Day et al. [34], the accuracy of the response scale as well as using a rather nonmetric variable as a metric one has to be discussed at this point. To the best of our knowledge, however, we are not aware of any questionnaire or single item that standardizes tanning behavior and records it in an equally brief manner. Since the reliability of measuring solar UVR exposure in our study has to be critically discussed, future studies should use validated questionnaires for measurement, such as the recently published validated “*Sun Exposure and Protection Index*” [31]. The major strength of our study is the comprehensive literature search and the content analysis of questionnaires used in already published studies. This ensures a reasoned selection of the SCSK Scale as an appropriate instrument to capture the level of knowledge. In addition, the translation by means of a standardized method involved two independent translators, both of whom have a degree in English linguistics, which is a state-of-the-art process.

## 5. Conclusion

The level of skin cancer and sun protection knowledge of German university students requires further careful investigation. Upcoming studies are needed to examine the level of knowledge as well as the association between the level of knowledge and appropriate protective behavior or tanning behavior, respectively. The psychometric properties of the SCSK require further thorough investigation. On the background of the literature overview and the first empirical experiences, it can be summarized that the SCSK in principle seems to be a suitable tool to assess the level of knowledge in terms of skin cancer and sun protection.

## Figures and Tables

**Table 1:** Overview of topics included in the questionnaires to assess knowledge about skin cancer and sun protection among students.

	Skin cancer			Risk factors							Ultraviolet radiation				Sun protection measures		
	Epidemiology	Signs or symptoms (e.g., moles and body spots)	Lethality and cure rate (e.g., SSE and therapy)	UVR exposure	Sunburn (incl. childhood sunburn)	Intentional (outdoor) tanning (incl. tanorexia)	Indoor tanning or sunbeds (incl. so-called benefits)	Heredity (incl. family history of skin cancer)	Skin phototype	Photosensitizing drugs	Peak hours and peak months	Further adverse effects (e.g., photoaging; not: sunburn)	Positive effects (e.g., vitamin D)	General knowledge (e.g., UVI and ozone layer)	Sun protection measures (e.g., shade, clothes, hat)	Sunscreen knowledge (e.g., on meaning of SPF)	Sunscreen usage (e.g., indication and application)
*References found in the initial literature search (December 31, 2018)*																	
Vail-Smith and Felts 1993 [42]				x	x	x						x			x		
Jerkegren et al. 1999 [43]		x	x	x								x	x	x		x	
Gillani et al. 2001 [44]	x	x	x	x				x	x								
Knight et al. 2002 [45]		x															
Cottrell et al. 2005 [46]	x	x	x	x	x			x	x					x			x
Hymowitz et al. 2006 [47]	x	x															
Dennis et al. 2009 [48]		x			x		x							x			
Patel et al. 2010 [49]											x				x		x
Felts et al. 2010 [26]					x	x					x						
Castilho et al. 2010 [50]				x				x									
Spradlin et al. 2010 [51]	x	x	x	x	x			x	x					x			x
Mahmoodabad et al. 2011 [52]	x	x	x					x			x	x		x	x		
Wołosik et al. 2012 [53]		x		x	x	x			x			x	x				
Isvy et al. 2013 [54]				x	x	x	x		x	x	x	x	x	x			
Day et al. 2013 [55]	x	x	x	x	x		x				x	x			x	x	x
Gao et al. 2014 [56]				x								x	x	x			
Yilmaz et al. 2015 [23]		x													x		
Zuba et al. 2016 [21]							x	x	x	x		x				x	
Othman Bahakim et al. 2016 [57]				x								x	x				
Rodriguez-Gambetta et al. 2016 [58]				x					x							x	x
Uğrlu et al. 2016 [59]				x	x							x					
Awadh et al. 2016 [60]																x	x
Urasaki et al. 2016 [61]		x		x					x		x						
Basch et al. 2017 [62]																x	x
Ivanov et al. 2018 [27]	x	x	x	x	x		x							x	x	x	
Celik et al. 2018 [28]	x	x	x	x	x		x	x	x							x	x
Haney et al. 2018 [63]^†^	x	x		x	x	x	x	x			x			x	x	x	x
Rasul et al. 2018 [64]				x	x							x	x				

*Additional references found in the lastly updated literature search (January 21, 2021)*																	
Almuqati et al. 2019 [20]				x	x		x				x		x			x	x
Iglesias-Puzas et al. 2019 [18]				x							x	x				x	x
Memon et al. 2019 [65]				x								x				x	
Ponce et al. 2019 [19]				x							x	x				x	x
Dallazem et al. 2019 [22]				x				x				x				x	x
Gunarić et al. 2019 [66]	x		x	x				x				x		x			
Byrne and Markham 2020 [29]	x	x			x											x	x
Kalil et al. 2020 [67]		x		x				x	x								

SPF = sun protection factor; SSE = skin self-examination; UVI = UV index. x = topic included in questionnaire. ^†^ = Haney et al. 2018 [63] used the Turkish version of the “Skin Cancer and Sun Knowledge Scale” in their study.

**Table 2:** Sociodemographic characteristics of the study sample.

Age	
Mean	26.25
SD	4.58
Range	19–46
Sex	*n* (%)
Male	15 (14.0)
Female	92 (86.0)
Self-assessed phototype according to the Fitzpatrick skin phototype classification system [40]	*n* (%)
Skin phototype I	1 (0.9)
Skin phototype II	34 (31.8)
Skin phototype III	54 (50.5)
Skin phototype IV	17 (15.9)
Skin phototype V	1 (0.9)
Skin phototype VI	0 (0)
Personal or family history of skin cancer	*n* (%)
Personal history of skin cancer	1 (0.9)
Family history of skin cancer	19 (17.8)
Nonexistent	82 (76.6)
I do not know	5 (4.7)
Currently enrolled program	*n* (%)
Bachelor	59 (55.1)
Master	48 (44.9)
Study course	*n* (%)
Health sciences	30 (28.0)
Nursing sciences	27 (25.2)
Cosmetic sciences	22 (20.6)
Electrical engineering	2 (1.9)
Metal technology	11 (10.3)
Ecotrophology	15 (14.0)
Completed apprenticeship	*n* (%)
Yes	84 (78.5)
No	23 (21.5)

**Table 3:** Participants' sun exposure and tanning behavior.

	Total sample (*n* = 107)	Female (*n* = 92)	Male (*n* = 15)
Sun exposure on weekdays	% (*n*)	% (*n*)	% (*n*)
(1) 30 minutes or less	(0)	(0)	0 (0)
(2) 31 minutes to 1 hour	41.1 (44)	38.0 (35)	60.0 (9)
(3) 2 hours	23.4 (25)	23.9 (22)	20.0 (3)
(4) 3 hours	0.9 (1)	1.1 (1)	0 (0)
(5) 4 hours	20.6 (22)	20.7 (19)	20.0 (3)
(6) 5 hours	8.4 (9)	9.8 (9)	0 (0)
(7) 6 hours	5.6 (6)	6.5 (6)	0 (0)
Sun exposure on weekends	% (*n*)	% (*n*)	% (*n*)
(1) 30 minutes or less	0.9 (1)	1.1 (1)	0 (0)
(2) 31 minutes to 1 hour	11.2 (12)	9.8 (9)	20.0 (3)
(3) 2 hours	0 (0)	0 (0)	0 (0)
(4) 3 hours	0 (0)	0 (0)	0 (0)
(5) 4 hours	31.8 (34)	30.4 (28)	40.0 (6)
(6) 5 hours	27.1 (29)	26.1 (24)	33.3 (5)
(7) 6 hours	29.0 (31)	32.6 (30)	6.7 (1)
Sun exposure (combined and weighted score)			
Mean	4.05	4.16	3.39
SD	1.39	1.41	1.01
Tanning behavior	% (*n*)	% (*n*)	% (*n*)
Never	0 (0)	0 (0)	0 (0)
Once or twice a year	23.4 (25)	21.7 (20)	33.3 (5)
3–5 times a year	29.0 (31)	27.2 (25)	40.0 (6)
7–12 times a year	25.2 (27)	27.2 (25)	13.3 (2)
More than once a month	22.4 (24)	23.9 (22)	13.3 (2)

**Table 4:** Total number of correctly answered items of the “Skin Cancer and Sun Knowledge Scale.”

Item		Total	Female	Male
		(*n* = 107)	(*n* = 92)	(*n* = 15)
		% (*n*)	% (*n*)	% (*n*)
1	I should stay out of the sun if my shadow is shorter than my body.	36.4 (39)	35.9 (33)	40.0 (6)
2	Sunbathing for only a couple of weeks a year (e.g., when on holiday) increases your likelihood of getting skin cancer.	60.7 (65)	60.9 (56)	60.0 (9)
3	Solariums/sunbeds are a safe way to get a tan.	96.3 (103)	96.7 (89)	93.3 (14)
4	When using sunscreen, you can tan without any negative effects.	86.0 (92)	88.0 (81)	73.3 (11)
5	Having a tan protects my skin from the sun.	62.6 (67)	64.1 (59)	53.3 (8)
6	A *fake/spray on tan* provides me with no protection from the sun.	85.0 (91)	85.9 (79)	80.0 (12)
7	Keeping your skin tanned at a solarium during the winter protects it from sun damage during the summer	85.0 (91)	87.0 (80)	73.3 (11)
8	Gradual tanning eliminates most of the negative effects of lengthy exposure to the sun.	28.0 (30)	30.4 (28)	13.3 (2)
9	A tan is a sign that the skin is damaged.	10.3 (11)	10.9 (10)	6.7 (1)
10	UVR (ultraviolet ray) from tanning beds is safer than UVR from the sun.	77.6 (83)	79.3 (73)	66.7 (10)
11	Tanning is an unsafe way to get the vitamin D your body needs.	32.7 (35)	34.8 (32)	20.0 (3)
12	A tan is a sign of good health.	77.6 (83)	80.4 (74)	60.0 (9)
13	If you are not usually exposed to the sun, being severely sunburned two or three times during your life will probably not increase your chances of skin disease.	66.4 (71)	68.5 (63)	53.3 (8)
14	The only way a person can get skin cancer is from too much exposure to the sun.	87.9 (94)	85.9 (79)	100 (15)
15	People with dark skin cannot get skin cancer.	95.3 (102)	95.7 (88)	93.3 (14)
16	When should sunscreen be applied for best protection?	97.2 (104)	97.8 (90)	93.3 (14)
17	How often should SPF 30 sunscreen be reapplied?	60.7 (65)	64.1 (59)	40.0 (6)
18	When is the sun the strongest?	88.8 (95)	88.0 (81)	93.3 (14)
19	Damage caused by the sun can be repaired by:	61.7 (66)	66.3 (61)	33.3 (5)
20	What type of clothing usually blocks more UV radiation (from the sun)?	21.5 (23)	21.7 (20)	20.0 (3)
21	What does SPF 30 mean?	47.7 (51)	45.7 (42)	60.0 (9)
22	Can you get a sunburn?	86.9 (93)	85.9 (79)	93.3 (14)
23	Which of the following increases your risk of skin cancer?	72.9 (78)	72.8 (67)	73.3 (11)
24	What is the most common form of skin cancer?	8.4 (9)	9.8 (9)	0 (0)
25	Which of the following could be a sign of skin cancer?	29.9 (32)	28.3 (26)	40.0 (6)

## Data Availability

The datasets used and analyzed during the current study are available from the corresponding author on reasonable request.

## Abbreviations

BCC: Basal cell carcinoma
ESS: European Social Survey
GDPR: General Data Protection Regulation
IARC: International Agency for Research on Cancer
KC: Keratinocyte carcinoma
KR-20: Kuder-Richardson 20 formula
*M*: Mean
Max: Maximum
Min: Minimum
MM: Malignant melanoma
NMSC: Nonmelanoma skin cancer
SC: Skin cancer
SCC: Squamous cell carcinoma
SCSK: Skin Cancer and Sun Knowledge Scale
SD: Standard deviation
SEPI: Sun Exposure and Protection Index
SPF: Sun protection factor
SSE: Skin self-examination
TRAPD: Questionnaire translation process: translation, review, adjunction, pretest, and documentation
UVR: Ultraviolet radiation.

## Supplementary Materials

Supplementary Materials Additional File 1: Study characteristics of studies assessing knowledge among university students. Title of data: Study characteristics of studies assessing skin cancer and sun protection knowledge among university students.
