# Supplementary material for: UV-Induced Skin Cancer Knowledge, Sun Exposure, and Tanning Behavior among University Students: Investigation of an Opportunity Sample of German University Students
Source: J Skin Cancer. 2021 Dec 29;2021:5558694. doi: 10.1155/2021/5558694 (PMC8731302; doi:10.1155/2021/5558694)
Supplement: Supplementary Materials — Additional File 1: Study characteristics of studies assessing knowledge among university students. Title of data: Study characteristics of studies assessing skin cancer and sun protection knowledge among university students. [file 5558694.f1.docx]

**Additional File 1:** Study characteristics of studies assessing knowledge among university students.

**Title of data:** Study characteristics of studies assessing skin cancer and sun protection knowledge among university students.

| t | **Country** | **Study population** | **Sample Size** | **Male** % (n) | **Female** % (n) | **Age** (mean) | **Age** (SD) |
| --- | --- | --- | --- | --- | --- | --- | --- |
| Vail-Smith & Felts 1993 [42] | USA | University students (*not specified*) | 296 | 39.2 (116) | 60.8 (180) | N/A | N/A |
| Jerkegren et al. 1999 [43] | Sweden | University students (*various fields of education*) | 296 | 53.04 (157) | 46.96 (139) | 24.2 | N/A |
| Gillani et al. 2001 [44] | Pakistan | Medical students | 71 | 66.20 (47) | 33.80 (24) | 20.30 | N/A |
| Knight et al. 2002 [45] | USA | University students (*not specified*) | 489 | 30.06 (147) | 69.94 (342) | N/A | N/A |
| Cottrell et al. 2005 [46] | USA | College students (*various fields of education*) | 453 | 46.6 (211) | 53.4 (242) | 19.2 | N/A |
| Hymowitz et al. 2006 [47] | USA | Medical students | 238 | 62.00 (148) | 38.00 (90) | N/A | N/A |
| Dennis et al. 2009 [48] | USA | College students (*not specified*) | 162 | 27 (44) | 73 (118) | N/A | N/A |
| Patel et al. 2010 [49] | USA | Medical students | 270 | 56.70 (152) | 43.30 (116) | 23.50 | 2.80 |
| Felts et al. 2010 [26] | USA | College students (*not specified*) | 596 | 36.90 (219) | 63.10 (375) | N/A | N/A |
| Castilho et al. 2010 [50] | Brazil | University students (*various fields of education*) | 368 | 45.1 (166) | 54.9 (202) | 22.10 | 5.2 |
| Spradlin et al. 2010 [51] | USA | University students (various fields of education) | 492 | 47.2 (232) | 51.4 (260) | N/A | N/A |
| MahmoodAbad et al. 2011 [52] | Iran | University students (*various fields of education*) | 230 | 27.80 (64) | 72.20 (166) | 21.00 | 2.60 |
| Wołosik et al. 2012 [53] | Poland | Pharmacy students & cosmetology students | 132 | N/A | N/A | N/A | N/A |
| Isvy et al. 2013 [54] | France | Medical students | 570 | 30.10 (172) | 69.90 (398) | N/A | N/A |
| Day et al. 2013 [55] | Australia | University students (*not specified*) | 162 | 0 (0) | 100 (162) | 20.53 | 2.32 |
| Gao et al. 2014 [56] | China | Medical students | 385 | 40.5 (156) | 59.5 (229) | 21.25 | N/A |
| Yilmaz et al. 2015 [23] | Turkey | Nursing students | 1.178 | 23.85 (281) | 76.15 (897) | N/A | N/A |
| Zuba et al. 2016 [21] | Poland | Medical students | 190 | 20.50 (39) | 79.40 (191) | 22.30 | 2.40 |
| Othman Bahakim et al. 2016 [57] | Saudi Arabia | Non-medical university students (*various fields of education*) | 399 | 50.1 (200) | 49.9 (199) | 21.0 | 1.82 |
| Rodriguez-Gambetta et al. 2016 [58] | Peru | Medical students | 299 | 36.80 (110) | 63.20 (189) | N/A | N/A |
| Uğrlu et al. 2016 [59] | Turkey | University students (*various fields of education*) | 404 | 37.6 (152) | 62.4 (252) | 21.00 | 2.34 |
| Awadh et al. 2016 [60] | Malaysia | Pharmacy students & medical students | 161 | 36.60 (59) | 63.40 (102) | N/A | N/A |
| Urasaki et al. 2016 [61] | Brazil | University students (*not specified*) | 385 | 39.3 (151) | 60.70 (234) | 21.80 | N/A |
| Basch et al. 2017 [62] | USA | University students (*not specified*) | 315 | 39.05 (123) | 60.95 (192) | N/A | N/A |
| Ivanov et al. 2018 [27] | USA | Osteopathic medical students | 121 | 47.10 (57) | 52.90 (64) | 24.10 | 2.00 |
| Celik et al. 2018 [28] | Turkey | Nursing students | 965 | 20.40 (197) | 79.60 (768) | 20.85 | 1.87 |
| Haney et al. 2018 [63]† | Turkey | Nursing students | 376 | 17.80 (67) | 82.20 (309) | 21.56 | 1.96 |
| Rasul et al. 2018 [64] | Iraq | University students (*not specified*) | 413 | 58.60 (242) | 41.40 (171) | N/A | N/A |
| Almuqati et al. 2019 [20] | Saudi Arabia | Non-medical female university students | 501 | 0 (0) | 100 (501) | 21.23 | 1.24 |
| Iglesias-Puzas et al. 2019 [18] | Spain | Nursing students | 200 | 14.50 (28) | 85.50 (172) | 23.23 | 4.90 |
| Memon et al. 2019 [65] | Pakistan | Medical students | 597 | 26.1 (156) | 73.90 (441) | 20.70 | 1.6 |
| Ponce et al. 2019 [19] | Spain | Medical students | 286 | 31.11 (89) | 68.88 (197) | 21.30 | 3.40 |
| Dallazem et al. 2019 [22] | Brazil | University students (*various fields of education*) | 371 | 45.80 (170) | 54.20 (201) | 22.46 | 5.09 |
| Gunarić et al. 2019 [66] | Bosnia & Herzegovina | University students (*medical students and non-healthcare-related faculty students*) | 140 | 35.70 (50) | 64.30 (90) | 20.60 | 1.70 |
| Byrne & Markham 2020 [29] | Ireland | Medical students | 312 | 34.73 (108) | 65.27 (203) | N/A | N/A |
| Kalil et al. 2020 [67] | Brazil | Medical students | 250 | 30.40 (76) | 69.60 (174) | 21.93 | 3.76 |
| **Abbreviations:** N/A = not available. | | | | | | | |
